# Modeling community integration in workers with delayed recovery from mild traumatic brain injury

**DOI:** 10.1186/s12883-015-0432-z

**Published:** 2015-10-09

**Authors:** Tatyana Mollayeva, Colin M. Shapiro, Shirin Mollayeva, J. David Cassidy, Angela Colantonio

**Affiliations:** Graduate Department of Rehabilitation Science, Collaborative Program in Neuroscience, University of Toronto, Toronto, Canada; Toronto Rehab-University Health Network, Ontario, Canada; Toronto Western Hospital, University Health Network, Ontario, Canada; Youthdale Child & Adolescent Sleep Clinic, Ontario, Canada; Faculty of Arts and Science, University of Toronto, Toronto, Canada; Aquired Brain Injury Research Lab, University of Toronto, Toronto, Canada; Division of Epidemiology, Dalla Lana School of Public Health, University of Toronto, Toronto, Canada; Department of Sport Science and Clinical Biomechanics, Faculty of Health, University of Southern Denmark, Odense, Denmark; Department of Occupational Science and Occupational Therapy, University of Toronto Ontario, Ontario, Canada

**Keywords:** Insomnia, Traumatic brain injury, Concussion, Recovery, Community integration, Diagnostic modeling

## Abstract

**Background:**

Delayed recovery in persons after mild traumatic brain injury (mTBI) is poorly understood. Community integration (CI) is endorsed by persons with neurological disorders as an important outcome. We aimed to describe CI and its associated factors in insured Ontario workers with delayed recovery following mTBI.

**Methods:**

A cross-sectional study of insured workers in the chronic phase following mTBI was performed at a rehabilitation hospital in Ontario, Canada. Sociodemographic, occupational, injury-related, clinical, and claim-related data were collected from self-reports, medical assessments, and insurers’ referral files. Community Integration Questionnaire (CIQ) scores were compared using analysis of variance or Spearman’s correlation tests. Stepwise multivariable linear regression models were used to evaluate the associations with CI.

**Results:**

Ninety-four workers with mTBI (45.2 ± 9.9 years old, 61.2 % male) at 197 days post-injury (interquartile range, 139–416 days) were included. The CIQ total and subscale scores were similar to those reported in more severe TBI samples. The CIQ scores were moderately to strongly correlated with various sociodemographic, claim-related, and clinical variables. In the multivariable regression analysis, several covariates accounted for 36.4 % of the CIQ variance in the final fully adjusted model.

**Discussion:**

This study evaluated CI in workers with mTBI, and analyzed its associated variables. Analysis revealed insomnia, head or neck pain, being married or in a relationship, time since injury, and a diagnosis of possible/probable malingering were independently associated with limited CI.

**Conclusions:**

Workers with delayed recovery from mTBI experience difficulty with CI. Insomnia is a particularly relevant covariate, explaining the greater part of its variance. To enhance participation, care should focus on clinical and non-clinical covariates.

**Electronic supplementary material:**

The online version of this article (doi:10.1186/s12883-015-0432-z) contains supplementary material, which is available to authorized users.

## Background

Traumatic brain injury (TBI) is a serious neurological disorder [1–3], with variable outcomes that include significant morbidity [4–7] and a decreased ability to function in society [5–9]. Moderate, severe, and penetrating TBIs are associated with the most adverse effects [9–14], although the effects of mild TBI (mTBI) have recently received increased attention, as approximately 75 % of all TBIs are due to mild or concussive events [15–18]. Many persons with mTBI recover fully, usually within days or weeks [18, 19], although 15–23 % of patients experience disabling symptoms that persist beyond 3 months [20, 21].

Many of these symptoms are not specific to TBI [22], and while the list is long, insomnia, the inability to sleep adequately at night given the opportunity [23],has been recognized as extremely important for explaining many of these symptoms in the general population, including cognitive disturbances, dizziness, fatigue, depression, and pain [24, 25].

When confronted with persistent symptoms long after the injury, most relevant parties (i.e., clinicians, insurers, and claim adjudicators) are not aware of their indicators [26]. Therefore, unnecessary clinical and diagnostic investigations may be ordered to assist the parties in their decision making. These investigations typically focus on variables that are derived from three predictive models for adverse mTBI outcomes. However, when the Transforming Research and Clinical Knowledge in TBI (TRACK-TBI) study was performed at three American medical centers to externally validate these models [27], researchers reported that two clinical models [28, 29] had minimal ability to discriminate between patients with favorable and non-favorable outcomes. The third model [30], featuring education, extracranial injury, and levels of post-concussion symptoms(i.e., depression, pain) as predictors of full return to work at 6 months post injury, could not be validated because of missing data. A focus on identifying more specific outcome measures was suggested for future research [27].

Recent initiatives have emphasized the importance of patients’ perceptions when assessing neurological outcomes [31]. In this context, the most relevant outcomes include family comfort, economic and social participation [32], falling under community integration (CI) concept [33]. Therefore, post-mTBI CI may be useful for measuring injury outcomes [34].

**Fig. 1:**
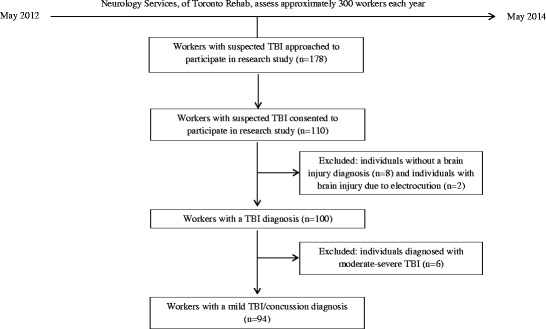
Flow chart depicting process of selection of participating individuals’ data for analysis. *TBI* Traumatic brain injury

Given the complexity of CI, we developed a model of CI for persons with TBI to investigate the following hypotheses among workers with delayed recovery from mTBI (Fig. 1): (1) CI would be poor; (2) insomnia would be negatively associated with CI; (3) previously reported clinical and claim-related variables [27–30] would be associated with CI; and (4) previously unexplored psychosocial variables (i.e., family relationship, personality traits) would be associated with CI.

## Methods

This study’s design was reviewed and approved by the Research Ethics Boards at the Toronto Rehab-University Health Network (UHN) and the University of Toronto. The findings were reported in compliance with the Transparent Reporting of a Multivariable Prediction Model for Individual Prognosis or Diagnosis (TRIPOD) guidelines [35].

### Procedure and participants

Since 1998, the Neurology Service of the Toronto Rehab-UHN has provided exclusive expert diagnoses and treatment recommendations for Workplace Safety and Insurance Board (WSIB)-insured workers who have sustained a head trauma at work and have not returned to work within 3 months after the injury. The multidisciplinary team of specialists establishes a TBI diagnosis based on the initial loss of consciousness (LOC), Glasgow Coma Scale (GCS) score, post-traumatic amnesia (PTA), magnetic resonance imaging (MRI), and clinical assessment.

### Recruitment

**Fig. 2:**
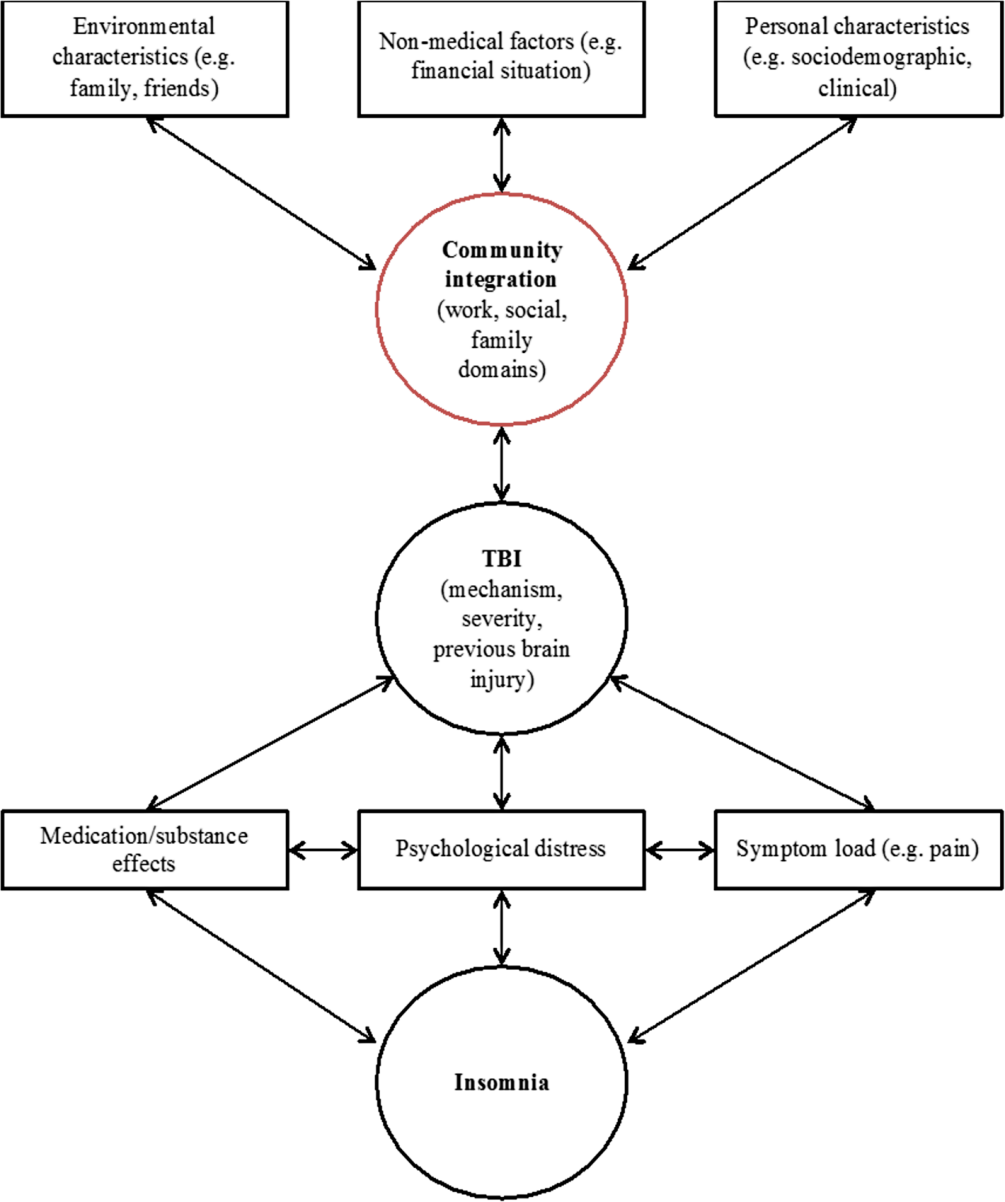
Conceptual framework of the construct of community integration in traumatic brain injury (TBI). Format adapted from Fayer & Hand [73]

All participants were recruited between May 2012 and May 2014. Initial contact was made with prospective participants (*n* = 178) at orientation sessions, where they were informed of the ongoing study and were invited to participate. The final sample included 110 consenting (in writing) participants, including 94 participants who were later diagnosed with mTBI/concussion (Fig. 2).

To compare our sample to a consecutive sample of workers, and to indirectly assess our sample’s representativeness, we performed a retrospective chart review of consecutive individuals (*n* = 294) who were referred for the same services and were assessed in the same clinic during 2003. No significant differences were observed in injury severity, sex, and age. Non-significant (*p* >0.05) differences were observed in working status and marital status at the time of assessment. Our sample contained more people who were working part- or full-time at their assessment, and fewer people who were single, widowed, or divorced. To maintain sample homogeneity, our analyses only utilized data for participants who were diagnosed with mTBI (*n* = 94).

### Instruments and measures

The clinical and diagnostic data included injury severity, presence of LOC, retrograde or anterograde PTA, and neuroimaging data. Clinical and claim-related variables were also collected from the participants’ medical and WSIB files, and included previous disability claims, employer-employee relations, WSIB-worker relations, and cases of malingering (DSM-IV-TR Axis IV) [36]. Occupational variables were gathered from insurer files (i.e., Employers’ Report of Injury/Illness Form 7), and included the workers’ occupation and weekly salary at the time of injury. A detailed description of the instruments that were used is presented in Additional file 1: Table S1, and the studied variables are presented in Additional file 2: Table S2.

The Community Integration Questionnaire (CIQ) [37] total score was used to measure outcome. All self-reported data were collected at the same time as the outcome assessment. The diagnostic investigations and clinical assessments were performed within a short period, during which no intervening treatments were commenced.

### Statistical analysis

SAS software (version 9.3, SAS Inc., Cary, NC) was used for all data analyses. Means and standard deviations or medians and ranges were used to describe continuous data, and frequency counts were used to describe categorical data. Continuous data were tested for normality before the analysis. Spearman’s correlation coefficients (r_s_) were used to examine the associations between the CIQ total score and the continuous variables. A one-way analysis of variance was used to assess the associations between the CIQ total score and the categorical explanatory variables with two or more levels.

We built four regression models for the dependent variable (CIQ total score), grouping previously reported predictors and *a priori* hypotheses into: (1) sociodemographic, (2) clinical, (3) claim-related, and (4) injury-related models. To limit collinearity and ensure parsimonious models, Spearman’s correlation coefficients were calculated for the clinically-relevant associations between covariates. The strongest correlations were observed between depression and insomnia (r_s_: 0.56), and between pain and insomnia (r_s_: 0.38). Stepwise eliminations were performed using p-values of ≥0.05 as the limiting threshold. Sex and age were included in every model, regardless of any association. Data for two participants who were injured >10 years earlier were omitted. The final regression model was derived using variables that were significantly associated with CI in the four individual models. The final regression analysis indicated that the 92 participants provided sufficient power for this study, with approximately nine participants per independent variable [38, 39].

## Results

Table 1 presents the characteristics of the 94 participants (58 men and 36 women) with a clinical diagnosis of mTBI. The mean age was 45.2 ± 9.9 years. Twenty-five workers (27 %) were single, widowed, or divorced, and 56 workers (60 %) had at least a post-secondary degree. Thirty-three workers (35 %) were in the middle or low weekly income categories at their injury.

**Table 1:** Socio-demographic, injury-related, clinical, and claim-related characteristics of the study population

Category	Variables	n (%N^a^)	Mean (SD)/median (Q3-Q1) (continuous variables)	CIQ score mean (SD) (binary/categorical variables)^b^	Rho (continuous variables)^b^	*P*-value
Socio-demographic	Sex					
	Male	58 (62)	NA	13.96 (4.67)	NA	0.086
	Female	36 (38)		15.56 (6.01)		
	Age, years	94 (100)	45.2 (9.94)	NA	−0.092	0.377
	Marital status					
	Married/common law	69 (73)	NA	13.81 (4.63)	NA	0.018
	Single/divorced/widowed	25 (27)		16.68 (6.18)		
	English first language					
	Yes	77 (82)	NA	14.82 (4.85)	NA	0.079
	No	17 (18)		12.47 (6.66)		
	Education					
	≤High school	34 (36)	NA	11.21 (3.28)	NA	0.006
	High school-college, prof. diploma	32 (34)		14.45 (5.78)		
	University and higher	24 (27)		17.20 (4.89)		
	Weekly income, $CAD	94 (100)	1056 (510)	NA	0.189	0.074
Injury-related	Time since injury, days	94 (100)	197 (416-139)	NA	−0.166	0.110
	Mechanism of injury					
	* Struck by inanimate object*					
	Yes	18 (19)	NA	12.67 (4.33)	NA	0.084
	No	76 (81)		15.03 (5.32)		
	* Struck by another person*					
	Yes	10 (11)		17.10 (4.89)		0.105
	No	84 (89)		14.27 (5.19)		
	* Struck against object/structure*					
	Yes	16 (17)		16.62 (4.81)		0.247
	No	78 (83)		14.38 (5.23)		
	* Fall*					
	Yes	18 (19)		13.61 (5.09)		0.386
	No	76 (81)		14.80 (5.25)		
	Loss of consciousness	86 (100)^a^				
	Yes	29 (31)	NA	14.17 (6.14)	NA	0.572
	No	56 (69)		14.86 (4.78)		
	Post-traumatic amnesia	86 (100)^a^				
	Yes	21 (25)	NA	13.62 (6.00)	NA	0.235
	No	65 (75)		15.15 (4.81)		
	Previous head trauma	90 (100)^a^				
	Yes	23 (25)	NA	15.70 (5.12)	NA	0.299
	No	67 (75)		14.39 (5.21)		
	Trauma-related head MRI findings	84 (100)^a^				
	Yes	0	NA	NA	NA	NA
	No	84 (100)				
Clinical	Comorbid conditions by self-report					
	* Arthritis*	93 (100)^a^				
	Yes	34 (37)	NA	15.05 (5.24)	NA	0.868
	No	59 (63)		13.59 (5.09)		
	* Diabetes mellitus*					
	Yes	5 (5)		10.40 (3.78)		0.065
	No	89 (95)		14.81 (5.20)		
	* Heart disease*					
	Yes	6 (6)		13.17 (8.13)		0.057
	No	88 (94)		14.67 (5.01)		
	Number of comorbid conditions	94 (100)	2.22 (1.04)	NA	−0.216	0.037
	DSM-IV-TR disorders					
	* Adjustment disorder*	88 (100)^a^				
	Yes	45 (51)	NA	13.80 (5.62)	NA	0.265
	No	43 (49)		15.05 (4.75)		
	* Anxiety disorder*					
	Yes	40 (45)		13.08 (5.49)		0.028
	No	48 (55)		15.52 (4.76)		
	* Mood disorder*					
	Yes	37 (42)		13.92 (6.14)		0.456
	No	51 (58)		14.75 (4.57)		
	* Personality traits*	92 (100)^a^				
	* Cluster B*					
	Yes	15 (17)		9.87 (3.16)		<0.0001
	No	77 (83)		15.50 (5.11)		
	* Cluster C*					
	Yes	42 (47)		13.50 (5.66)		0.034
	No	50 (53)		15.88 (4.47)		
	* Sleep disorder*					
	Yes	9 (10)		17.71 (4.27)		0.977
	No	79 (90)		17.79 (6.23)		
	* Substance-related disorder*					
	Yes	13 (15)		12.69 (5.07)		0.200
	No	75 (85)		14.71 (5.22)		
	Comorbid conditions, by scales					
	Anxiety (HADS-A)	NA	10.71 (4.74)	NA	−0.317	0.002
	Depression (PHQ-9)		16.77 (6.67)		−0.320	0.002
	Insomnia (ISI)		17.46 (6.07)		−0.370	<0.001
	Pain (VAS-P), current		5.02 (2.40)		−0.344	<0.001
	Symptom load					
	* Balance issues*					
	Yes	44 (47)	NA	14.82 (4.91)	NA	0.673
	No	50 (53)		14.36 (5.51)		
	* Bodily pain*					
	Yes	32 (34)		13.53 (5.59)		0.164
	No	62 (66)		15.11 (4.97)		
	* Mood disturbance*					
	Yes	62 (66)		14.19 (4.86)		0.327
	No	32 (34)		15.31 (5.84)		
	* Head and/or neck pain*					
	Yes	87 (93)		14.22 (5.19)		0.019
	No	7 (7)		19.00 (3.83)		
	* Photo-/phonophobia*					
	Yes	14 (15)		15.07 (6.63)		0.701
	No	80 (85)		14.49 (4.97)		
Claim- related	* Current working status*	94 (100)^a^				
	Working full-/part time	40 (43)	NA	17.78 (6.00)	NA	0.607
	On disability/laid off	54 (57)		16.45 (7.33)		
	* Previous WSIB claims*	88 (100)^a^				
	Yes	8 (9)		16.12 (5.59)		0.332
	No	80 (91)		14.24 (5.19)		
	* Probable/possible malingering, by DSM-IV-TR*					
	Yes	14 (16)		10.93 (4.68)		0.006
	No	74 (84)		15.07 (5.08)		

^a^ *N* = 94 unless otherwise specified

^b^Community integration scores were compared using analysis of variance or Spearman’s correlation tests

*NA* Not applicable

Time since injury (TSI) distribution was greatly skewed (median, 197 days post-injury [interquartile range, 139–416 days]). The major mechanisms of injury were falls (19.1 %), being struck by (19.1 %) or against (17 %) an object, motor vehicle incidents (12.8 %), and being struck by a person (10.5 %). Among the 86 workers with available LOC and PTA data, 31 % had experienced some degree of LOC and 24.7 % had experienced PTA. Eighty-four participants underwent MRI, and none exhibited trauma-related brain changes.

Most participants (57 %) were receiving disability benefits at the time of assessment, and the rest were working full- or part-time. The most common pre-morbid occupational categories were skilled or factory workers and machine operators or assemblers (44 %); elementary occupations (35 %); managerial, professional, associate professional, or technician positions (14 %); and clerical support, service, or sales workers (7 %). Forty-five workers (47.9 %) worked shifts at their injury; 38 (84 %) worked rotating shifts; and 7 (16 %) worked night shifts.

Substantial proportions of the workers had one or more DSM-IV TR diagnoses (Table 1), including anxiety (45.5 %), mood (42.1 %), somatoform (29 %), and substance-related (14.8 %) disorders. Nine workers (10.2 %) were diagnosed with a sleep disorder, including 8 with a sleep-related breathing disorder. The most common post-morbid symptoms that affected functioning were head and neck pain (92.6 %), cognitive (71.3 %), mood- (66 %), sleep- (63 %),and balance-related (47 %) disturbances, and bodily pain (34 %).

**Fig. 3:**
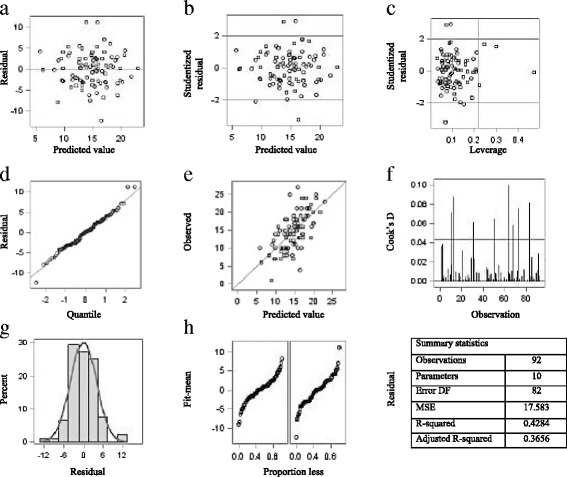
Fit diagnostics for community integration (**a**-**h**)

**Fig. 4:**
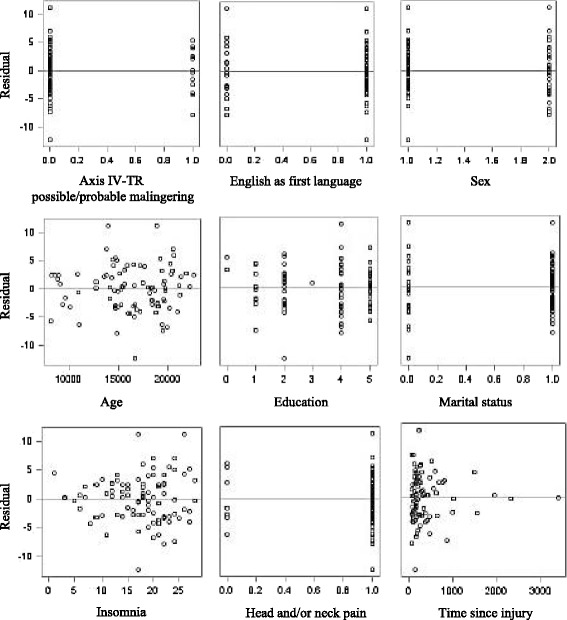
Residuals by regressor for community integration

The CIQ was normally distributed and its internal consistency was appropriate(Cronbach’s alpha, α = 0.70)(Figs. 3 and 4).

### Bivariate analyses

Single, widowed, or divorced workers had significantly higher CIQ total scores (*p* <0.001), compared to workers who were married or in relationships. Workers whose first language was English also had significantly higher CIQ scores (*p* = 0.006). In addition, participants who were employed as managers, professionals, technicians, or associate professionals had significantly higher CIQ scores (*p* = 0.014), compared to participants who worked in clerical support or service work, sales, and elementary occupations. Significant differences were observed in the CIQ scores for workers who did and did not report head and neck pain (*p* = 0.005). Workers with Axis IV-TR diagnoses of possible/probable malingering (*p* <0.001), cluster B disorders (*p* = 0.004), cluster C disorders (*p* = 0.009), mood disorders (*p* = 0.049), and cognitive disorders (*p* = 0.004) had significantly lower CIQ scores. The CIQ scores were also negatively correlated with pain (*p* <0.001), anxiety (*p* = 0.009), depression (*p* <0.001), and insomnia (*p* <0.001).

### Multivariable regression analyses

**Fig. 5:**
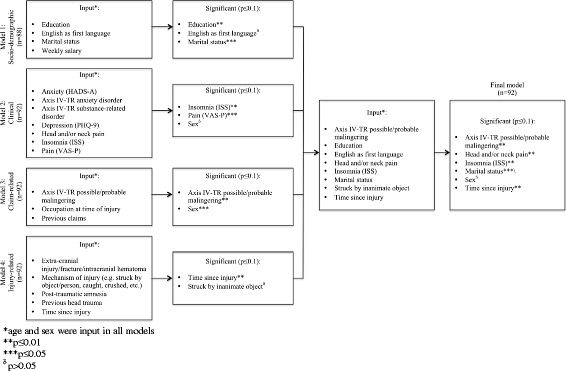
Depiction of multivariate regression analysis for community integration

Four preliminary multivariate linear regression analyses were performed to evaluate associations with CI. All models were age- and sex-adjusted (Fig. 5).

The final regression model included education, marital status, English as the first language, TSI, being struck by inanimate objects, insomnia, head and/or neck pain, and Axis IV-TR malingering as independent variables. After the stepwise selection, the final model explained 36.6 % of the CI variance, and contained five significant variables: insomnia (β = –0.250, *p* <0.001), Axis IV-TR malingering (β = –4.923, *p* <0.001), TSI (β = –0.002, *p* = 0.025), head and/or neck pain (β = –4.186, *p* = 0.015), and marital status (β = –2.087, *p* = 0.048) (Table 2 and Fig. 5).

**Table 2:** Summary of the stepwise multiple regression analysis for the final model, sex and age adjusted

From model	Variable	β Coefficient	SE	*P* value	Partial R^2^	Model R^2^
*#1 Socio-demographic*	Education	0.380	0.323	0.242	0.011	0.011
	English as first language	1.304	1.179	0.272	0.018	0.029
	Marital status	−2.087	1.041	0.048	0.043	0.072
*#2 Clinical*	Insomnia	−0.250	0.076	<0.001	0.159	0.231
	Head and/or neck pain	−4.186	1.679	0.015	0.039	0.270
*#3 Claim-related*	Axis-IV-TR malingering	−4.923	1.302	<0.001	0.069	0.339
*#4 Injury- related*	Time since injury	−0.002	0.001	0.025	0.075	0.414
	Struck by inanimate object	1.965	1.059	0.076	0.014	0.428

## Discussion

In this study of 94 workers with mTBI, we found that insomnia, head or neck pain, being married or in a relationship, TSI, and a diagnosis of possible/probable malingering were independently associated with limited CI.

Unfortunately, there are no normative data for CIQ scores in mTBI. However, the CIQ total and subscale scores in the present study were similar to the mean scores at 1 year post-injury in participants with more severe TBI, as reported by Sanders et al. (GCS: 8.43 ± 3.8) [40] and Seale et al. (GCS: 6.5 ± 3.7) [41]. As in previous studies [42, 43], the highest scores were observed in the social integration domain and the lowest scores were observed in the productive activities domain. However, our participants had slightly higher scores in the home integration domain. One possible explanation for this discrepancy is that our study evaluated workers with persistent symptoms, and the majority of participants were receiving disability benefits at their time of assessment. Thus, our data suggests that disability status is strongly associated with impaired productive activity, although it had a lesser effect on social and family integration in our study.

Our findings also highlight the associations between insomnia, pain, and depression, in agreement with earlier reports [42–49]. However, insomnia has been viewed as a symptom that occurs in the context of another disorder (i.e., depression [42–45], pain [46–48], or brain injury [49]), rather than as an independent disorder within mTBI. In many depressed individuals, however, insomnia signals the onset of depression and may prevent recovery, even after adequate treatment [50, 51]. Similarly, the stress created by insomnia may aggravate or serve as a catalyst for the involvement of the hypothalamic orexinergic or hypocretinergic system in pain and headaches [52]. This may be relevant for our participants, as the majority continued to experience pain and depressive symptoms, despite being diagnosed with and treated for both at their assessment. Therefore, insomnia and its causes should be addressed separately from depression and pain in mTBI, in order to maximize treatment outcomes, especially regarding CI.

We also found that a diagnosis of possible/probable malingering was associated with a poorer CI. Although the presence of malingering or symptom exaggeration does not preclude the existence of symptoms or disorders, it does make the quantification of these issues impossible [53, 54]. Therefore, future research should be designed identify the determinants of a malingering diagnosis among injured workers. According to the DSM-IV, a diagnosis of malingering is appropriate when ≥2 of 4 criteria are met [36, 55]: (1) presentation of symptoms in a medico-legal context, (2) discordance between the individual’s stated disability and objective data, (3) uncooperative behavior during evaluation, and (4) presentation of antisocial personality disorder. However, our participants met the first two criteria, as they were referred by the WSIB for evaluation and mTBI is a clinical diagnosis that cannot be confirmed with objective data (i.e., none of our participants had positive MRI findings, and there is currently no sensitive and specific imaging technique to diagnose mTBI) [55, 56]. In addition, a worker with certain personality traits may find being questioned about their disability or injury to be unnerving, which may satisfy the third criterion independent of any malingering. Furthermore, recommendations have been published to implement neuropsychological testing for possible malingering in persons with TBI [57]. This raises the issue of language proficiency [49, 58], as these evaluations would be performed using tests that were developed in English and have not been validated in other languages. Therefore, English proficiency is an important variable to consider in future studies regarding performance validity.

We also found that a longer TSI was associated with a poorer CI after mTBI. Although this association was not evaluated longitudinally, our results may indicate that other relevant factors (e.g., psychological, medical, or other) can develop after the mTBI and strengthen over time, thereby impeding CI [59]. Similarly, psychological and psychosocial factors that present in the early post-injury period can influence outcomes across the entire recovery timeline [60]. Unfortunately, the cross-sectional nature of our study does not allow us to provide insight regarding this topic. Nonetheless, our results support the notion that patient assessments should begin as early as possible, which can establish baseline findings for CI and time-dependent outcomes.

Unexpectedly, being married was not related to a better CI, and our results indicate that marriage had a negative effect on CI. In contrast, earlier studies have reported that married people with a disability have higher levels of life satisfaction [61, 62], fewer handicaps [63], and longer life expectancy [64], compared to their unattached counterparts. However, our results should be interpreted with caution, as one-third of our participants were single, divorced, or widowed at the time of assessment, and we did not assess whether or not this status changed after their injury. Nevertheless, our findings suggest that workers who are married or in relationships may rely on their partners for assistance in performing their family and societal duties, which would hinder their independent CI. This observation highlights uncertainty regarding the role of marriage and/or partner support in the context of compromised CI, and longitudinal analysis of relationship status and patient outcomes (both personal and familial) may provide insight regarding their interdependence and independence.

The strengths of our study include a well-characterized sample of insured workers with a confirmed mTBI diagnosis. In addition, this is the first study to simultaneously address the prevalence of various sociodemographic, clinical, claim-related, and TBI-related variables, and their subsequent impact on CI. Furthermore, we utilized the TRIPOD checklist, which is a valuable reference for good reporting of multivariable prediction models. Therefore, the present study provides foundational data for a comprehensive longitudinal study that can evaluate the risk factors for prolonged recovery and reduced post-mTBI CI.

Our study has several limitations. First, our model for estimating CI was complex, which reflects the large number of covariates that were associated with CI and were not included in our final model. Therefore, a study with a large sample, standardized data collection and calculations will be needed to validate our model. Second, generalization of our findings may be limited, as a high prevalence of clinically relevant disorders was observed in our sample, and each of these disorders have been associated with poor post-injury outcomes. Furthermore, our study only aimed to evaluate workers who had a prolonged recovery after their injury, as they experience the greatest effects of mTBI and are the most difficult to rehabilitate. Third, the R^2^ value of 0.366 for our final regression analysis indicates that that only 36. 6 % of the variance in the mean CI can be explained by the above named variables and insomnia explains most of the variance (i.e., 15.9 %). This finding may be due to the omission of information regarding various critical CI areas, such as psychological sense of community, satisfaction with community, and perception of safety [65–67]. However, given that our results support the notion of CI as a time-dependent construct, and our data consisted of various time series, the R2 value for insomnia provides solid support of it as a covariate of CI, bearing in mind the fact that we are looking for meaningful associations in the context of delayed recovery from mTBI in the presence of many *a priori* defined relationships and a relatively small sample size.

Finally, this study highlighted the factors that were associated with CI in a population of workers with mTBI, although the longitudinal relationships between these factors and poor post-injury outcomes remain to be determined. Therefore, further research regarding this topic may facilitate the development of interventions that improve the CI of injured workers with mTBI.

## Conclusions

Community integration is increasingly being recognized as a highly relevant outcome in outpatient populations, and is currently listed among the criteria that are used to assess the participation domain of the International Classification of Functioning, Disability, and Health [68] after TBI. Our results suggest that the CI may differ across various clinical populations, based on the presence or absence of insomnia and head or neck pain. Therefore, specialists who assess workers with mTBI should be particularly sensitive to these complaints, and should thoroughly investigate the etiology of these symptoms. In addition, we found that marital status may hinder CI, and that CI was related to an Axis IV-TR diagnosis of malingering. Thus, efforts to increase post-injury CI should be guided by a comprehensive understanding of the diverse factors that contribute to outcomes beyond the persistent post-concussive symptoms.

## Additional files

Additional file 1: Table S1. Standardized measured used, with examples of uses in TBI populations and their evaluated applicability [69–72]. (DOCX 45 kb) Additional file 2: Table S2. Categories and types of variables collected. (DOCX 16 kb)

## Abbreviations

CIQ: Community integration questionnaire
GCS: Glasgow coma scale
HADS-A: Hospital anxiety and depression scale
LOC: Loss of consciousness
MRI: Magnetic resonance imaging
mTBI: Mild traumatic brain injury
MVI: Motor vehicle injury
PHQ-9: Patient health questionnaire-9
PTA: Post-traumatic amnesia
P-VAS: Pain visual analogue scale
RTW: Return to work
SD: Standard deviation
TSI: Time since injury
TBI: Traumatic brain injury
TRIPOD: Transparent reporting of a multivariable prediction model for individual prognosis or diagnosis
UHN: University health network
WSID: Workplace safety and insurance board
