# Supplementary material for: Modeling community integration in workers with delayed recovery from mild traumatic brain injury
Source: BMC Neurol. 2015 Oct 9;15:194. doi: 10.1186/s12883-015-0432-z (PMC4600293; doi:10.1186/s12883-015-0432-z)
Supplement: Additional file 1: Table S1. — Standardized measured used, with examples of uses in TBI populations and their evaluated applicability [69–72]. (DOCX 45 kb) [file 12883_2015_432_MOESM1_ESM.docx]

| **Measure** | **Descriptive**   - Purpose - Content - Response options - Recall period - Access | **Practical application**   - Cost to obtain - Administration - Scoring/interpretation - Respondent burden - Materials, admin burden | **Psychometric information**   - Development - Acceptability - Reliability/Validity/Responsiveness | **Critical appraisal of value to mTBI** **population**   - Strengths, cautions - Clinical, research application |
| --- | --- | --- | --- | --- |
| Patient health questionnaire-9 (PHQ-9) [1] | - Assessment of depression severity - 9 items (i.e. depressive symptoms) - 4-point Likert scale; 0: not at all, 3: nearly every day - Past two weeks - Public | - No cost; online at: [www.uspreventiveservicestaskforce.org/Home/GetFileByID/218](http://www.uspreventiveservicestaskforce.org/Home/GetFileByID/218) - Items scored 0-27; total score =sum of 9 items; higher score=more severe depression; major depression if $\geq$5 symptoms are present more than half the days; other depression if 2 to 4 symptoms are present more than half the days, and one is a cardinal symptom (i.e. depressed mood or anhedonia); suicidality symptom counts regardless of duration - ≤5 min (variable, depends of patient characteristics) - Pen(cil) and paper; no training; scoring by researcher/clinician ≤5 min | - Module from the full PHQ, validated in primary care and obstetrics/gynecology clinics [1-3] - Validated also: TBI (mild, mod, sev) (n=135) [4]: - Sn and sp maximized when $\geq$5 PHQ-9 symptoms are present, with $\geq$1 a cardinal symptom (sn=93%, sp=89%, +ve predictive value (PPV)=63%, -ve predictive value (NPV)=99%, +ve likelihood ration=8.58, -ve likelihood ratio=.08, κ=.69) - PHQ-9 total cut-off score allowed differentiation of persons with and without major depression (sn=85%, sp=94%) - Convergent validity: correlations with other depression measures: *20-item Hopkins symptom checklist depression subscal*e *(SCL-20)* (r=.9, p<.001); *Hamilton rating scale for depression* (r=.78, p<.001); correlations with other health measures: *functional impairment item* (r=.59, p<.001); *general health perception* (r=.4, p<.001) - Discriminant validity: correlation with *head injury symptom checklist* (r=.49, p=.002 for symptom botherness; r=.44, p=.005 for symptom frequency) - Test-retest reliability: measure readministered within ≤7 days (r=.76, p<.001 for total score) - Injury severity did not alter sn and sp | - Quick, minimal effort for brain injured persons with difficulty concentrating [4] - Valid and reliable screening tool for detecting major depressive disorder in TBI |
| Hospital anxiety and depression scale (HADS-A) [5] | - Intensity of anxiety - 7 items - 4-point Likert scale, 0: not at all, 3: very often - In the past week - Not publicly accessible | - NA; contact: info@gl-assessment.co.uk - Self-administered - Total score is mean; higher score=more anxiety - Recommended cutoffs are: 8-10: mild cases, 11-15: moderate cases and 16 or above: severe cases - ≤5min (variable, depends on patient characteristics) - Pen(cil) and paper; no training; scoring by researcher/clinician ≤5 min | - Developed with input from patients and the physicians treating them; items generated by compiling questions relevant to generalized anxiety or depression; item reduction after completion by patients, examination by physician, and interviews by researchers [5] - Validated in TBI [6,7] - TBI (mild, moderate, severe) (n=123) [6]: - HADS-A vs SCID diagnosis: Cronbach’s alpha=.90, area under curve=.86 (CI:.80-.93), sn=81%, sp=79% | - Yields states of anxiety and emotional distress; looks at many aspects of anxiety - Relatively unaffected by psychiatric illness - Demonstrated validity as screening measure of anxiety in TBI of all severities |
| Insomnia severity index (ISI) [8,9] | - Severity of insomnia - 7 items (featured difficulty falling asleep, maintaining sleep, level of interference of these difficulties with perceived daytime functioning, the degree to which others notice sleep difficulties impact, and the level of distress/worry about sleep difficulties) - 5-point Likert scale; 0: not at al, 4: very much; 4 multiple choice questions - Past 2 weeks - Public | - No cost; online at: http://www.deploymentpsych.org/system/files/member_resource/Insomnia%20Severity%20Index%20-ISI.pdf - Self-administered - Scored 0-28, 0-7:no clinically significant insomnia, 8-14:sub-threshold insomnia, 15-21:clinical insomnia (moderate severity), 22-28: clinical insomnia (severe); higher score=greater fatigue - ≤5min (variable, depends on patient characteristics) - Pen(cil) and paper; no training; scoring by researcher/clinician ≤5 min | - To evaluate and screen for insomnia in general population - Used in brain injury [10] - Current study: mTBI (n=94): - Cronbach’s alpha=0.86 - ISI total score seven items as a unit (α=0.86); - Item-total score r range=0.57-0.83; - Individual item-item r range 0.19-0.71 | - Easy administration; brief; useful as a screening device or outcome measure - High internal consistency, as measured by Cronbach’s alpha - Scores may be impacted by poor perception in severe TBI |
| Community integration questionnaire (CIQ) [17] | - Assessment of social role limitations and community and community integration for people with ABI - 15 items across 3 domains (i.e. home, social, and productive activities integrations) - Most item range 0 to 2 points: yourself alone, yourself and someone else, someone else - Not specified - Public | - No cost; online at: http://www.rehabmeasures.org/Lists/RehabMeasures/DispForm.aspx?ID=894&Source=http%3A%2F%2Fwww%2Erehabmeasures%2Eorg%2Frehabweb%2Fallmeasures%2Easpx%3FPageView%3DShared - Self-administered - Total score 0-29; home integration(10), social (12), productive (7); higher score= better independence and community integration - <10 min - Pen(cil) and paper; no training; scoring by researcher/clinician ≤5 min | - In brain injury of various severity- mild, moderate, severe [12-14]: - TBI normative scores, by previous group order: mild- not known; moderate-severe-admission 11.6±4.2; discharge 16.3±5.3 [15], 14.26±5.52 [16] - Internal consistency: Cronbach’s alpha=0.76 [11] - Item-total correlations=0.32-0.67 [11] - Subtotal to total correlations =0.54-0.74, 0.79 for productivity, social, home integration [17] - Criterion validity: home, social, productive activity integration as rated by family member=0.17-0.45) - Content validity: excellent; 3 factors obtained by factor analysis mirrored experts [17] - Responsiveness: change over time (before/after rehabilitation [15,17]; pre-morbid-post morbid ratings [18] | - Recommended to use for outpatient and home care by TBI Taskforce ( TBI EDGE) - Developed to assess the social role limitations and community interaction of people with TBI - Woman may have higher total score/home integration sub scores; older persons lower scores - Cut-off scores not established |
| Visual analogue scale for pain (VAS-P) [18] | - Assessment of pain - 3 items (featured in current, best, and worse) - Continuum, 10   cm line (e.g. from “not at all” to “extremely”)   - Past 24 hours - In original article [18] | - In [18] - Self-administered - Score for each scale measured with ruler along 100 mm line to mark made by respondent; total score 0-100; higher score on fatigue items and lower score on vigor items=greater fatigue - <10 min - Pen(cil) and paper; minimal training; scoring by researcher/clinician ≤10 min | - Valid, reliable tool incorporating visual analogue scales to evaluate changes in pain over time period [18] - In brain injury: mTBI (n=90) vs trauma controls (n=80)[19]: - Mean/median VAS-P results mTBI at 1 week: 20.63/13 (21.58); controls: 22.49/15 (21.90); P=0.366 - Mean/median VAS-P results mTBI at 3 mos: 7.44/2 (13.08); controls: 7.49/1 (14.73); P=0.643 | - Quick completion; little bias because continuum; inter-rater reliability important because of objective nature in scoring - Valid, reliable measure in healthy persons; used in mTBI |

References

1. [Kroenke K](http://www-ncbi-nlm-nih-gov.myaccess.library.utoronto.ca/pubmed?term=Kroenke%20K%5BAuthor%5D&cauthor=true&cauthor_uid=11556941), [Spitzer RL](http://www-ncbi-nlm-nih-gov.myaccess.library.utoronto.ca/pubmed?term=Spitzer%20RL%5BAuthor%5D&cauthor=true&cauthor_uid=11556941), [Williams JB](http://www-ncbi-nlm-nih-gov.myaccess.library.utoronto.ca/pubmed?term=Williams%20JB%5BAuthor%5D&cauthor=true&cauthor_uid=11556941). The PHQ-9: Validity of a brief depression severity measure. J Gen Intern Med 2001;16(9):606-13.
2. Spitzer RL, Kroenke K, Williams JBW. Patient Health Questionnaire Study Group. Validity and utility of a self-report version of PRIME-MD: the PHQ Primary Care Study JAMA. 1999; 282:1737–44.
3. Spitzer RL, Williams JBW, Kroenke K. Validity and utility of the Patient Health Questionnaire in assessment of 3000 obstetric-gynecologic patients: the PRIME-MD Patient Health Questionnaire Obstetrics-Gynecology Study. Am J Obstet Gynecol 2000;183:759–69.
4. [Fann JR](http://www-ncbi-nlm-nih-gov.myaccess.library.utoronto.ca/pubmed?term=Fann%20JR%5BAuthor%5D&cauthor=true&cauthor_uid=16304487), [Bombardier CH](http://www-ncbi-nlm-nih-gov.myaccess.library.utoronto.ca/pubmed?term=Bombardier%20CH%5BAuthor%5D&cauthor=true&cauthor_uid=16304487), [Dikmen S](http://www-ncbi-nlm-nih-gov.myaccess.library.utoronto.ca/pubmed?term=Dikmen%20S%5BAuthor%5D&cauthor=true&cauthor_uid=16304487), [Esselman P](http://www-ncbi-nlm-nih-gov.myaccess.library.utoronto.ca/pubmed?term=Esselman%20P%5BAuthor%5D&cauthor=true&cauthor_uid=16304487), [Warms CA](http://www-ncbi-nlm-nih-gov.myaccess.library.utoronto.ca/pubmed?term=Warms%20CA%5BAuthor%5D&cauthor=true&cauthor_uid=16304487), [Pelzer E](http://www-ncbi-nlm-nih-gov.myaccess.library.utoronto.ca/pubmed?term=Pelzer%20E%5BAuthor%5D&cauthor=true&cauthor_uid=16304487), [Rau H](http://www-ncbi-nlm-nih-gov.myaccess.library.utoronto.ca/pubmed?term=Rau%20H%5BAuthor%5D&cauthor=true&cauthor_uid=16304487), [Temkin N](http://www-ncbi-nlm-nih-gov.myaccess.library.utoronto.ca/pubmed?term=Temkin%20N%5BAuthor%5D&cauthor=true&cauthor_uid=16304487). Validity of the patient health questionnaire-9 in assessing depression following traumatic brain injury. J Head Trauma Rehabil 2005; 20(6):501-11.
5. Zigmond AS, Snaith RP. The hospital anxiety and depression scale. Acta Psychiatrica Scandinavica 1983; 67 (6): 361–70.
6. Schwarzbold ML, Diaz AP, Nunes JC, Sousa DS, Hohl A, Guarnieri R, Linhares MN, Walz R. [Validity and screening properties of three depression rating scales in a prospective sample of patients with severe traumatic brain injury.](http://www-ncbi-nlm-nih-gov.myaccess.library.utoronto.ca/pubmed/24770656) Rev Bras Psiquiatr 2014;36(3):206-12.
7. Dahm J, Wong D, Ponsford J. [Validity of the Depression Anxiety Stress Scales in assessing depression and anxiety following traumatic brain injury.](http://www-ncbi-nlm-nih-gov.myaccess.library.utoronto.ca/pubmed/23830002)J Affect Disord 2013;151(1):392-6.
8. Bastien CH, Vallieres A, Morin CM. Validation of the Insomnia Severity Index as an outcome measure for insomnia research. Sleep Med 2001: 297–307.
9. Zeitzer JM, Friedman L, O’Hara R. Insomnia in the context of traumatic brain injury. J Rehabil Res Dev, 2009; 46: 827–36.
10. Mollayeva T, Kendzerska T, Colantonio A. [Self-report instruments for assessing sleep dysfunction in an adult traumatic brain injury population: a systematic review.](http://www-ncbi-nlm-nih-gov.myaccess.library.utoronto.ca/pubmed/23706309) Sleep Med Rev 2013;17(6):411-23.
11. [Burleigh SA](http://www-ncbi-nlm-nih-gov.myaccess.library.utoronto.ca/pubmed?term=Burleigh%20SA%5BAuthor%5D&cauthor=true&cauthor_uid=9426858), [Farber RS](http://www-ncbi-nlm-nih-gov.myaccess.library.utoronto.ca/pubmed?term=Farber%20RS%5BAuthor%5D&cauthor=true&cauthor_uid=9426858), [Gillard M](http://www-ncbi-nlm-nih-gov.myaccess.library.utoronto.ca/pubmed?term=Gillard%20M%5BAuthor%5D&cauthor=true&cauthor_uid=9426858). Community integration and life satisfaction after traumatic brain injury: long-term findings. J [Am J Occup Ther](http://www-ncbi-nlm-nih-gov.myaccess.library.utoronto.ca/pubmed/?term=Burleigh+SA%2C+Farber+R+S.%2C+et+al.+(1998).+%22Community+integration+and+life+satisfaction+after+traumatic+brain+injury%3A+long-term+findings.%22+Am+J+Occup+Ther+52(1)%3A+45-52.) 1998; 52(1):45-52.
12. [Cusick CP](http://www-ncbi-nlm-nih-gov.myaccess.library.utoronto.ca/pubmed?term=Cusick%20CP%5BAuthor%5D&cauthor=true&cauthor_uid=10745189), [Gerhart KA](http://www-ncbi-nlm-nih-gov.myaccess.library.utoronto.ca/pubmed?term=Gerhart%20KA%5BAuthor%5D&cauthor=true&cauthor_uid=10745189), [Mellick DC](http://www-ncbi-nlm-nih-gov.myaccess.library.utoronto.ca/pubmed?term=Mellick%20DC%5BAuthor%5D&cauthor=true&cauthor_uid=10745189). Participant-proxy reliability in traumatic brain injury outcome research. [J Head Trauma Rehabil](http://www-ncbi-nlm-nih-gov.myaccess.library.utoronto.ca/pubmed/?term=Cusick%2C+C.+P.%2C+Gerhart%2C+K.+A.%2C+et+al.+(2000).+%22Participant-proxy+reliability+in+traumatic+brain+injury+outcome+research.%22+J+Head+Trauma+Rehabil+15(1)%3A+739-749.) 2000; 15(1):739-49.
13. [Hall KM](http://www-ncbi-nlm-nih-gov.myaccess.library.utoronto.ca/pubmed?term=Hall%20KM%5BAuthor%5D&cauthor=true&cauthor_uid=11245760), [Bushnik T](http://www-ncbi-nlm-nih-gov.myaccess.library.utoronto.ca/pubmed?term=Bushnik%20T%5BAuthor%5D&cauthor=true&cauthor_uid=11245760), [Lakisic-Kazazic B](http://www-ncbi-nlm-nih-gov.myaccess.library.utoronto.ca/pubmed?term=Lakisic-Kazazic%20B%5BAuthor%5D&cauthor=true&cauthor_uid=11245760), [Wright J](http://www-ncbi-nlm-nih-gov.myaccess.library.utoronto.ca/pubmed?term=Wright%20J%5BAuthor%5D&cauthor=true&cauthor_uid=11245760), [Cantagallo A](http://www-ncbi-nlm-nih-gov.myaccess.library.utoronto.ca/pubmed?term=Cantagallo%20A%5BAuthor%5D&cauthor=true&cauthor_uid=11245760). Assessing traumatic brain injury outcome measures for long-term follow-up of community-based individuals. [Arch Phys Med Rehabil](http://www-ncbi-nlm-nih-gov.myaccess.library.utoronto.ca/pubmed/?term=Hall%2C+K.+M.%2C+Bushnik%2C+T.%2C+et+al.+(2001).+%22Assessing+traumatic+brain+injury+outcome+measures+for+long-term+follow-up+of+community-based+individuals.%22+Arch+Phys+Med+Rehabil+82(3)%3A+367-374.) 2001; 82(3):367-74.
14. Sherer M, Davis LC, Sander AM, Caroselli JS, Clark AN, Pastorek NJ. [Prognostic importance of self-reported traits/problems/strengths and environmental barriers/facilitators for predicting participation outcomes in persons with traumatic brain injury: a systematic review.](http://www-ncbi-nlm-nih-gov.myaccess.library.utoronto.ca/pubmed/24583024) Arch Phys Med Rehabil 2014; 95(6):1162-73.
15. [Seale GS](http://www-ncbi-nlm-nih-gov.myaccess.library.utoronto.ca/pubmed?term=Seale%20GS%5BAuthor%5D&cauthor=true&cauthor_uid=12455520), [Caroselli JS](http://www-ncbi-nlm-nih-gov.myaccess.library.utoronto.ca/pubmed?term=Caroselli%20JS%5BAuthor%5D&cauthor=true&cauthor_uid=12455520), [High WM Jr](http://www-ncbi-nlm-nih-gov.myaccess.library.utoronto.ca/pubmed?term=High%20WM%20Jr%5BAuthor%5D&cauthor=true&cauthor_uid=12455520), [Becker CL](http://www-ncbi-nlm-nih-gov.myaccess.library.utoronto.ca/pubmed?term=Becker%20CL%5BAuthor%5D&cauthor=true&cauthor_uid=12455520), [Neese LE](http://www-ncbi-nlm-nih-gov.myaccess.library.utoronto.ca/pubmed?term=Neese%20LE%5BAuthor%5D&cauthor=true&cauthor_uid=12455520), [Scheibel R](http://www-ncbi-nlm-nih-gov.myaccess.library.utoronto.ca/pubmed?term=Scheibel%20R%5BAuthor%5D&cauthor=true&cauthor_uid=12455520). Use of community integration questionnaire (CIQ) to characterize changes in functioning for individuals with traumatic brain injury who participated in a post-acute rehabilitation programme. [Brain Inj](http://www-ncbi-nlm-nih-gov.myaccess.library.utoronto.ca/pubmed/?term=Use+of+the+Community+Integration+Questionnaire+(CIQ)+to+characterize+changes+in+functioning+for+individuals+with+traumatic+brain+injury+who+participated+in+a+post-acute+rehabilitation+programme) 2002;16(11):955-67.
16. [Sander AM](http://www-ncbi-nlm-nih-gov.myaccess.library.utoronto.ca/pubmed?term=Sander%20AM%5BAuthor%5D&cauthor=true&cauthor_uid=10527092), [Fuchs KL](http://www-ncbi-nlm-nih-gov.myaccess.library.utoronto.ca/pubmed?term=Fuchs%20KL%5BAuthor%5D&cauthor=true&cauthor_uid=10527092), [High WM Jr](http://www-ncbi-nlm-nih-gov.myaccess.library.utoronto.ca/pubmed?term=High%20WM%20Jr%5BAuthor%5D&cauthor=true&cauthor_uid=10527092), [Hall KM](http://www-ncbi-nlm-nih-gov.myaccess.library.utoronto.ca/pubmed?term=Hall%20KM%5BAuthor%5D&cauthor=true&cauthor_uid=10527092), [Kreutzer JS](http://www-ncbi-nlm-nih-gov.myaccess.library.utoronto.ca/pubmed?term=Kreutzer%20JS%5BAuthor%5D&cauthor=true&cauthor_uid=10527092), [Rosenthal M](http://www-ncbi-nlm-nih-gov.myaccess.library.utoronto.ca/pubmed?term=Rosenthal%20M%5BAuthor%5D&cauthor=true&cauthor_uid=10527092). The community integration questionnaire revisited: an assessment of factor structure and validity. [Arch Phys Med Rehabil](http://www-ncbi-nlm-nih-gov.myaccess.library.utoronto.ca/pubmed/?term=Sander%2C+A.+M.%2C+Fuchs%2C+K.+L.%2C+et+al.+(1999).+%22The+Community+Integration+Questionnaire+revisited%3A+an+assessment+of+factor+structure+and+validity.%22+Arch+Phys+Med+Rehabil+80(10)%3A+1303-1308.) 1999; 80(10):1303-8.
17. Willer B, Ottenbacher KJ, Coad ML. [The community integration questionnaire. A comparative examination.](http://www-ncbi-nlm-nih-gov.myaccess.library.utoronto.ca/pubmed/8148099) Am J Phys Med Rehabil. 1994; 73(2):103-11.
18. Corrigan JD. [Community integration following traumatic brain injury.](http://www-ncbi-nlm-nih-gov.myaccess.library.utoronto.ca/pubmed/24525321) NeuroRehabilitation 1994;4(2):109-21.
19. Huskisson EC. Measurement of pain. Lancet 1974; 2: 1127–31.
