# Supplementary material for: Modeling community integration in workers with delayed recovery from mild traumatic brain injury
Source: BMC Neurol. 2015 Oct 9;15:194. doi: 10.1186/s12883-015-0432-z (PMC4600293; doi:10.1186/s12883-015-0432-z)
Supplement: Additional file 2: Table S2. — Categories and types of variables collected. (DOCX 16 kb) [file 12883_2015_432_MOESM2_ESM.docx]

| Category | Type | Variable (standardized measure used, where applicable) |
| --- | --- | --- |
| Injury-related | Continuous | Time since injury |
|  | Categorical | Mechanism of injury |
|  | Binary | LOC |
|  |  | PTA |
|  |  | Injury-related MRI findings |
| Socio-demographic | Continuous | Age (years) |
|  |  | Weekly salary at injury |
|  | Categorical | Education |
|  | Binary | Sex |
|  |  | Marital status |
|  |  | English as first language |
| Occupational | Categorical | Occupation at injury |
|  |  | Working status at assessment |
| Clinical | Continuous | Depression (PHQ-9) |
|  |  | Anxiety (HADS-A) |
|  |  | Insomnia (ISI) |
|  |  | Pain (P-VAS) |
|  | Binary | DSM-IV TR disorders |
|  |  | Previous head trauma |
|  |  | Clinically relevant comorbid conditions |
|  |  | Symptom load impacting functioning |
| Claim-related  Social  Family-related | Continuous | Community integration (CIQ) |
|  |  | Previous WSIB claims |
|  |  | Malingering (multidisciplinary assessment) |
